# Interleukin 27 deficiency drives dilated cardiomyopathy by ferroptosis

**DOI:** 10.1002/ctm2.70269

**Published:** 2025-03-21

**Authors:** Yan Zhao, Jing Dai, Angwei Gong, Sheng Jin, Chengjian Guan, Keke Wang, Qianli Ma, Haijuan Hu, Yuming Wu, Bing Xiao

**Affiliations:** ^1^ Department of Cardiology the Second Hospital of Hebei Medical University Shijiazhuang China; ^2^ Department of Clinical Diagnostics Hebei Medical University Shijiazhuang China; ^3^ Department of Physiology Hebei Medical University Shijiazhuang China; ^4^ The Key Laboratory of Neural and Vascular Biology Ministry of Education Shijiazhuang China; ^5^ Hebei Key Laboratory of Cardiovascular Homeostasis and Aging Shijiazhuang China

1

Dear Editor,

The molecular mechanisms underlying dilated cardiomyopathy (DCM) pathogenesis remain incompletely understood. In this study, we provide comprehensive evidence demonstrating that interleukin 27 (IL27) exerts protective effects in DCM by inhibiting ferroptosis, potentially opening new therapeutic options for DCM.

DCM features ventricular dilation with impaired cardiac function, demonstrating substantial morbidity and mortality. Despite therapeutic advancements, the 10‐year survival rate remains approximately 60%, emphasizing the urgent need for innovative therapeutic strategies.^1^ IL27, originating from immune cells, plays a key role in regulating the progression of various cardiovascular diseases.^2^ Recent clinical studies have indicated the cardiac tissues from DCM patients had higher IL27 mRNA levels, suggesting a potential link between IL27 and DCM.^3^ However, the precise mechanism through which IL27 influences DCM progression has remained elusive.

To establish a potential causal relationship between IL27 and DCM, we conducted Mendelian randomization analysis using genome‐wide association study data. The study design principles and framework are shown in Figure S1A,B, respectively. Through systematic analysis, we identified a previously unreported single nucleotide polymorphism (SNP), rs181209. The Wald ratio method suggested a significant inverse correlation between plasma IL27 levels and DCM risk (odds ratio 0.91, 95% confidence interval 0.84–0.98, *p* = .01) (Supplementary Figure 1C). This finding expanded upon previous research linking IL27 polymorphisms to DCM susceptibility, particularly the previously identified SNP rs153109.^4^ Notably, our identification of rs181209 provided new insights into the genetic architecture underlying IL27's cardioprotective effects and strengthened the evidence for a causal relationship between IL27 and DCM pathogenesis.

To validate our genetic findings and explore underlying mechanisms, we constructed multiple experimental models. First, we developed a doxorubicin (Dox)‐induced DCM model based on previous studies.^5^ Echocardiographic analysis revealed significant cardiac abnormities in Dox‐treated mice, characterized by reduced left ventricular ejection fraction and fractional shortening (LVEF and LVFS), thinning of both anterior and posterior left ventricular walls during systole (LVAWs and LVPWs), and enlarged left ventricular end‐systolic internal diameter (LVIDs) (Figure S2A–F). Histological analysis using Masson staining exposed a conspicuous rise in the myocardial fibrotic area (Figure S2G,H). Importantly, Dox treatment significantly decreased IL27 levels in both plasmas (Figure S2J) and cardiac tissue (Figure S2I,L), accompanied by increased IL27 receptor (IL27Ra) expression in cardiac tissue (Figure S2I,K), possibly reflecting a compensatory response to maintain IL27 signalling.

Given these findings, we generated two complementary genetic models: global IL27 knockout (IL27 KO) and cardiomyocyte‐specific IL27 receptor knockout (IL27Ra^ΔCM^) mice. This approach was informed by our observation that IL27Ra expression was mainly expressed in the heart of C57BL/6N mice (Supplementary Figure 3). The generation strategies for global and conditional knockouts are shown in Figures 1A and 2A, respectively. We confirmed knockout efficiency through PCR‐genotype identification and immunoblotting analyses (Figures 1B,C and 2B,C). While global IL27 deletion significantly reduced plasma IL27 levels (Figure 1D), cardiomyocyte‐specific IL27Ra deletion had no effect on circulating IL27 levels (Figure 2E). Both genetic models demonstrated cardiac dysfunction closely resembling the Dox‐induced phenotype. Echocardiographic analysis revealed significant reductions in LVEF and LVFS, ventricular wall thinning, and chamber dilation in both IL27 KO mice (Figure 1 E‐J) and IL27Ra^ΔCM^ mice (Figure 2D–J). Masson staining confirmed heightened fibrotic area in both models (Figures 1K,L and 2K,L). The above results suggested that IL27 deficiency would lead to DCM. Subsequently, we performed proteomic sequencing on cardiac tissues from both knockout models and their respective controls. Principal component analysis showed clear separation and tight clustering (Figure S4A,B). We identified 192 differentially expressed proteins in IL27 KO hearts (108 upregulated, 84 downregulated) and 99 differentially expressed proteins in IL27Ra^ΔCM^ hearts (53 upregulated and 46 downregulated). Notably, volcano plots (Figure S4C,D) and heatmaps (Figure S4E,F) showed consistent upregulation of ferritin heavy chain 1 in both datasets, suggesting ferroptosis as a key mechanism mediating IL27's effect in DCM pathogenesis.

**FIGURE 1:**
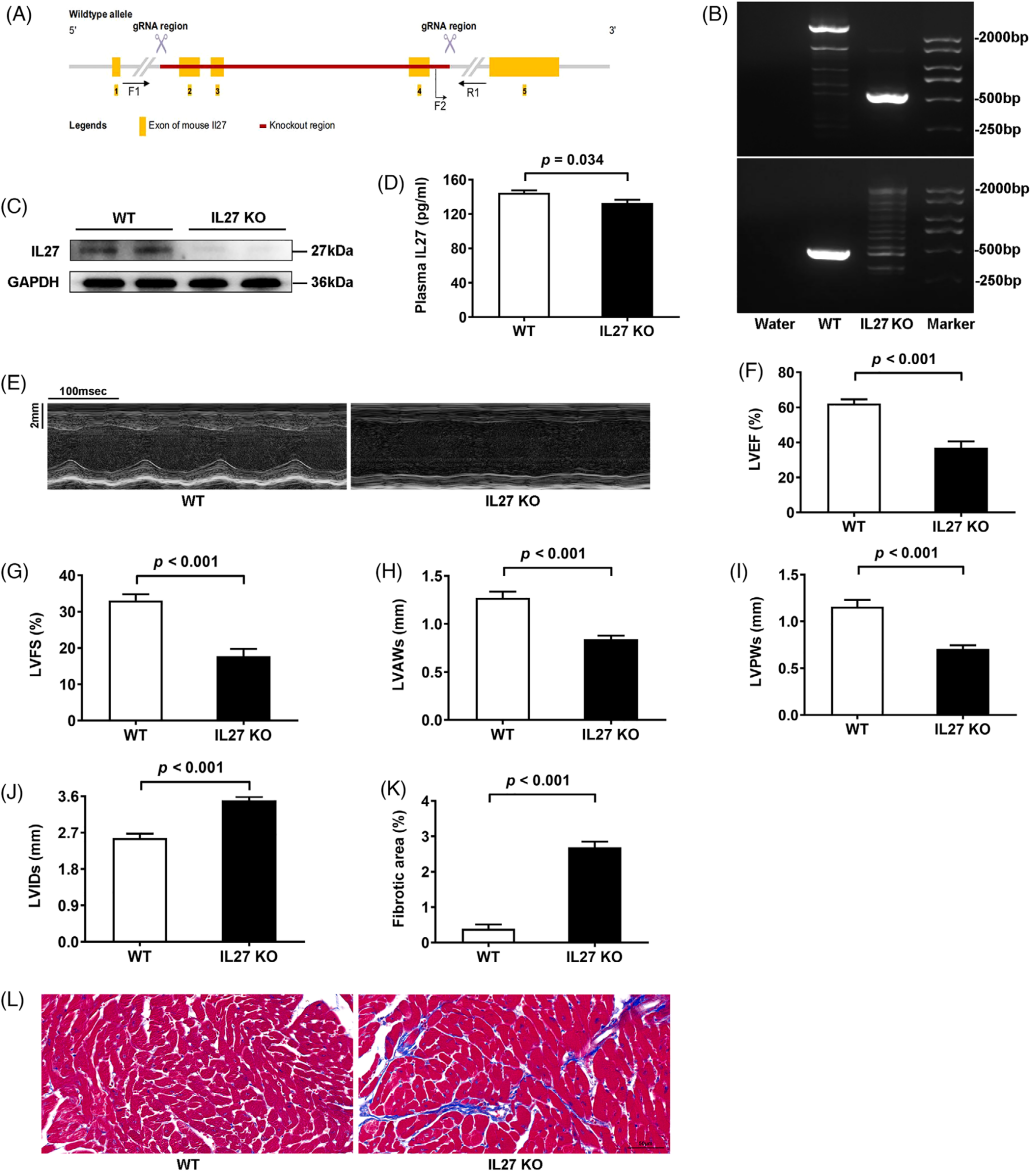
IL27 depletion induced DCM‐like phenotypes in mice. (A) Schematic illustration of traditional knockout of IL27. (B) Agarose gel image of PCR‐genotyping of genomic DNA. (C) Immunoblotting analysis of IL27 knockout efficiency (*n* = 2). (D) Quantitative analysis of plasma IL27 levels determined by ELISA (*n* = 6). (E) Representative images of M‐mode echocardiograms. Scale bar = 100 ms/2 mm. (F–J) Quantitative analysis of LVEF, LVFS, LVAWs, LVPWs and LVIDs (*n* = 6). (K–L) Representative images of Masson staining and quantitative analysis of myocardial fibrotic area (*n* = 6). Scale bar = 50 µm. Data are presented as mean ± SEM. WT: wildtype; IL27 KO: interleukin 27 knockout; DCM: dilated cardiomyopathy; LVEF: left ventricular ejection fraction; LVFS: left ventricular fractional shortening; LVAWs: left ventricular end‐systolic anterior wall thickness; LVPWs: left ventricular end‐systolic posterior wall thickness; LVIDs: left ventricular end‐systolic internal diameter. GAPDH: glyceraldehyde‐3‐phosphate dehydrogenase; ELISA: enzyme‐linked immunosorbent assay.

**FIGURE 2:**
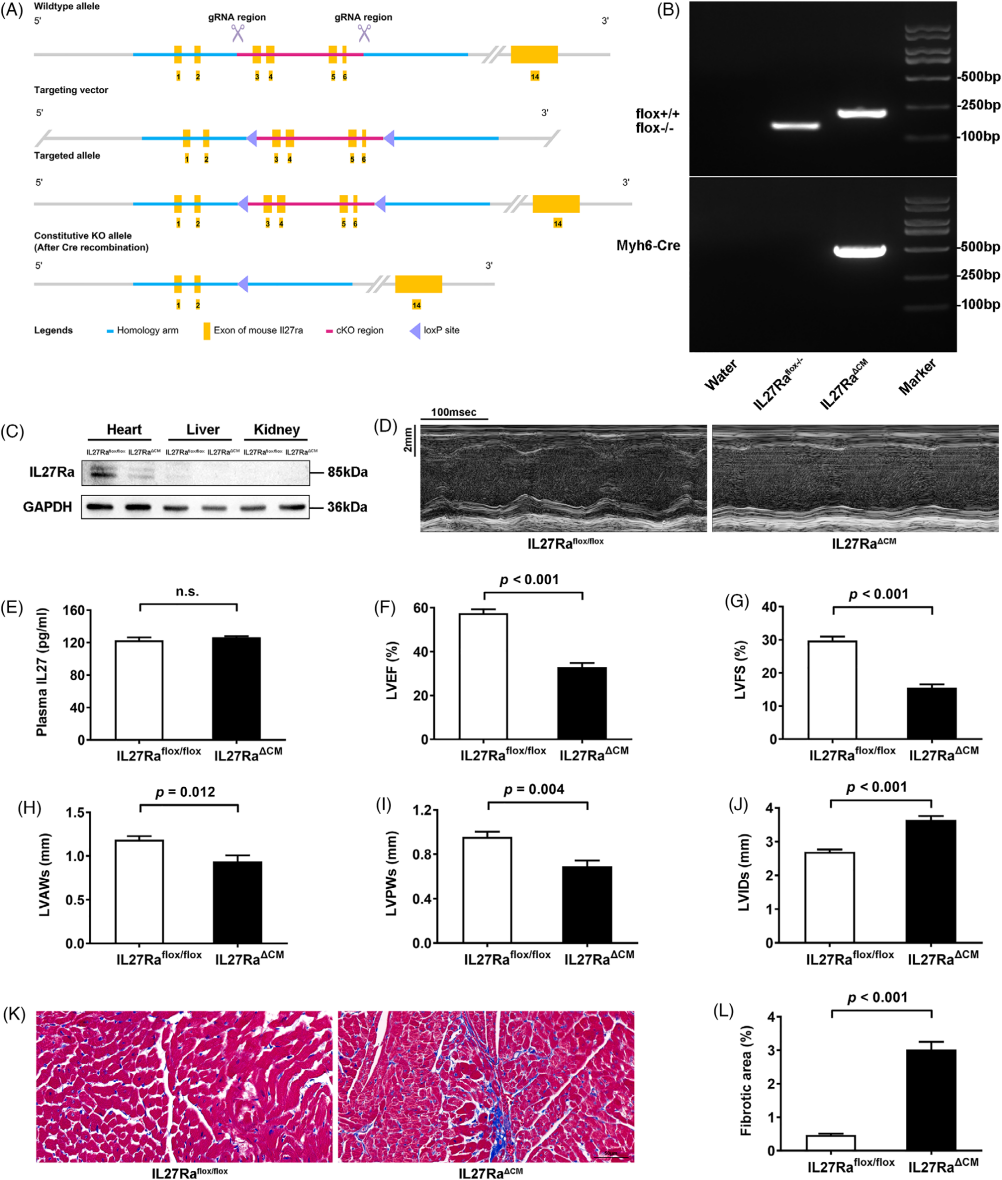
Cardiomyocyte‐specific knockout of IL27Ra led to DCM‐like phenotypes in mice. (A) Schematic diagram of generating IL27Ra^ΔCM^ mice by utilizing the Cre‐loxP recombination system. (B) Agarose gel image of PCR‐genotyping of genomic DNA. (C) Immunoblotting analysis of cardiomyocyte‐specific knockout efficiency of IL27Ra. (D) Representative images of M‐mode echocardiograms. Scale bar = 100 ms/2 mm. (E) Quantitative analysis of plasma IL27 levels determined by ELISA (*n* = 6). (F–J) Quantitative analysis of LVEF, LVFS, LVAWs, LVPWs and LVIDs (*n* = 6). (K–L) Quantitative analysis of fibrotic area and representative images of Masson staining (*n* = 6). Scale bar = 50 µm. Data are presented as mean ± SEM. IL27Ra^ΔCM^: cardiomyocyte‐specific IL27 receptor knockout; DCM: dilated cardiomyopathy; LVEF: left ventricular ejection fraction; LVFS: left ventricular fractional shortening; LVAWs: left ventricular end‐systolic anterior wall thickness; LVPWs: left ventricular end‐systolic posterior wall thickness; LVIDs: left ventricular end‐systolic internal diameter. GAPDH: glyceraldehyde‐3‐phosphate dehydrogenase; ELISA: enzyme‐linked immunosorbent assay.

To validate our proteomic findings, we assessed ferroptosis markers and key ferroptosis‐related proteins across all three animal models. Assessment of cardiac tissue in Dox‐treated mice revealed elevated total iron, and malondialdehyde levels, accompanied by reduced glutathione in cardiac tissues (Figure S5A–C), which was consistent with previous research.^6^ Besides, we observed downregulation of the transferrin receptor, coupled with upregulation of ferroportin and ferritin heavy chain 1 in Dox‐treated mice (Figure S5D–F). Similar changes were observed in both IL27 KO (Figure 3A–F) and IL27Ra^ΔCM^ mice (Figure 4A–F). Ferroptosis, characterized by iron accumulation and lipid peroxide generation, is tightly regulated by these proteins, which collectively maintain intracellular iron homeostasis by mediating iron import, export, and storage mechanisms.^7^, ^8^ These findings demonstrated a compensatory response to increased iron accumulation and oxidative stress which were hallmark features of ferroptosis. To establish the therapeutic relevance of these findings, we administered the specific ferroptosis inhibition ferrostatin‐1 across our experimental models. This intervention yielded significant improvements in cardiac function in Dox‐treated mice, evidenced by enhanced LVEF and LVFS, normalized ventricular wall thickness (LVAWs and LVPWs), and reduced chamber dilation (LVIDs) (Figure S5G,I–M), and reduced myocardial fibrosis (Figure S5H,N). Similar cardioprotective effects were observed in both IL27 KO (Figure 3G–N) and IL27Ra^ΔCM^ mice (Figure 4G–N), providing compelling evidence that inhibition of ferroptosis could effectively rescue cardiac dysfunction in the context of IL27 deficiency.

**FIGURE 3:**
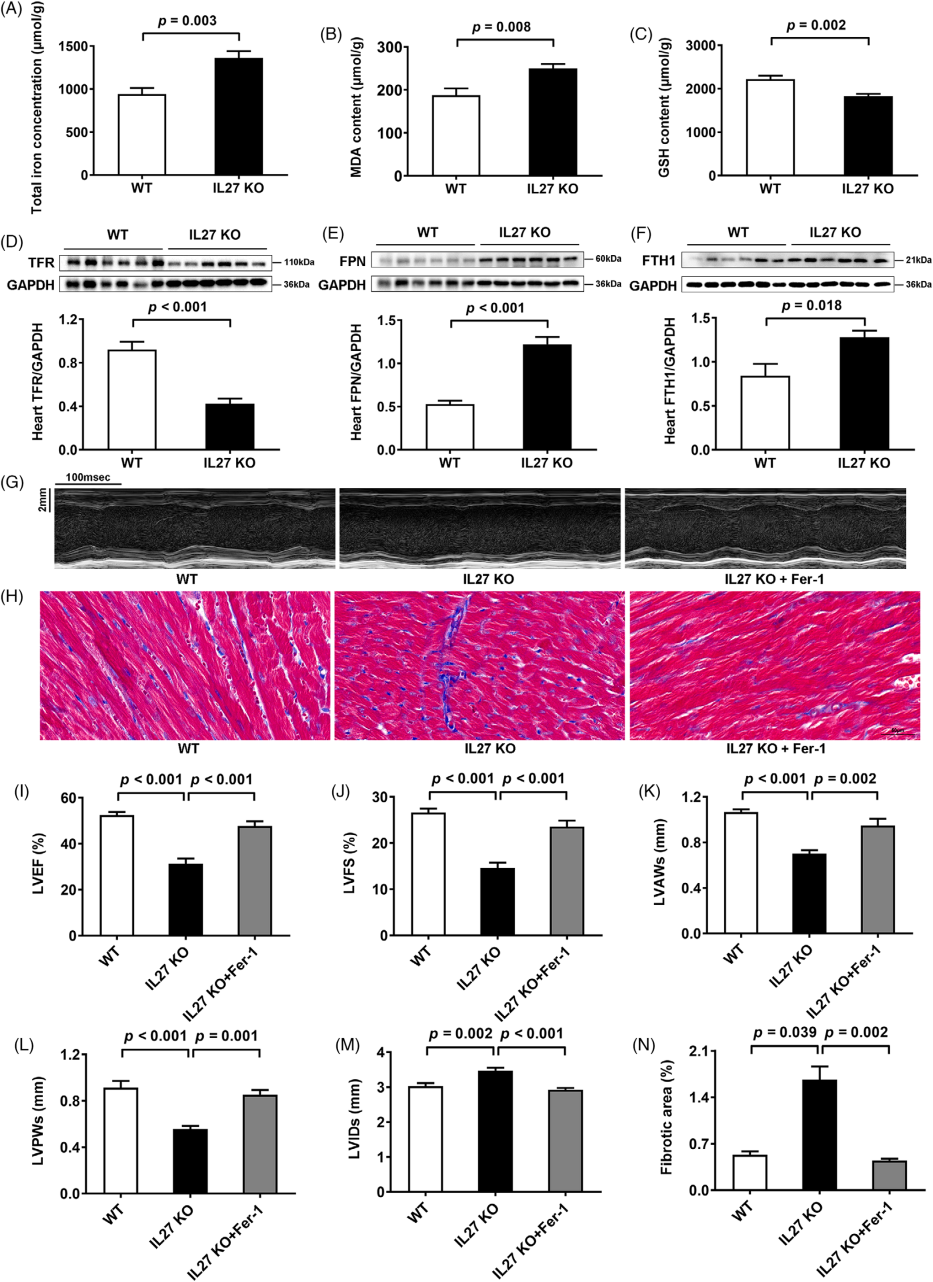
Inhibition of ferroptosis ameliorated DCM in IL27 KO mice. (A–C) Total iron, MDA and GSH levels of myocardial tissues in WT and IL27 KO groups (*n* = 6). (D–F) Immunoblotting results and quantitative analysis of TFR, FPN and FTH1 protein expression levels in WT and IL27 KO groups. (*n* = 6). (G) Representative images of M‐mode echocardiograms. Scale bar = 100 ms/2 mm. (H) Representative images of Masson staining and quantitative analysis of myocardial fibrotic area. Scale bar = 50 µm. (I–M) Quantitative analysis of LVEF, LVFS, LVAWs, LVPWs and LVIDs (*n* = 6). (N) Quantitative analysis of myocardial fibrotic area (*n* = 6). Data are presented as mean ± SEM. MDA: malondialdehyde; GSH: glutathione; TFR: transferrin receptor; FPN: ferroportin; FTH1: ferritin heavy chain 1; GAPDH: glyceraldehyde‐3‐phosphate dehydrogenase; WT: wildtype; IL27 KO: interleukin 27 knockout; Fer‐1: ferrostatin‐1; DCM: dilated cardiomyopathy; LVEF: left ventricular ejection fraction; LVFS: left ventricular fractional shortening; LVAWs: left ventricular end‐systolic anterior wall thickness; LVPWs: left ventricular end‐systolic posterior wall thickness; LVIDs: left ventricular end‐systolic internal diameter.

**FIGURE 4:**
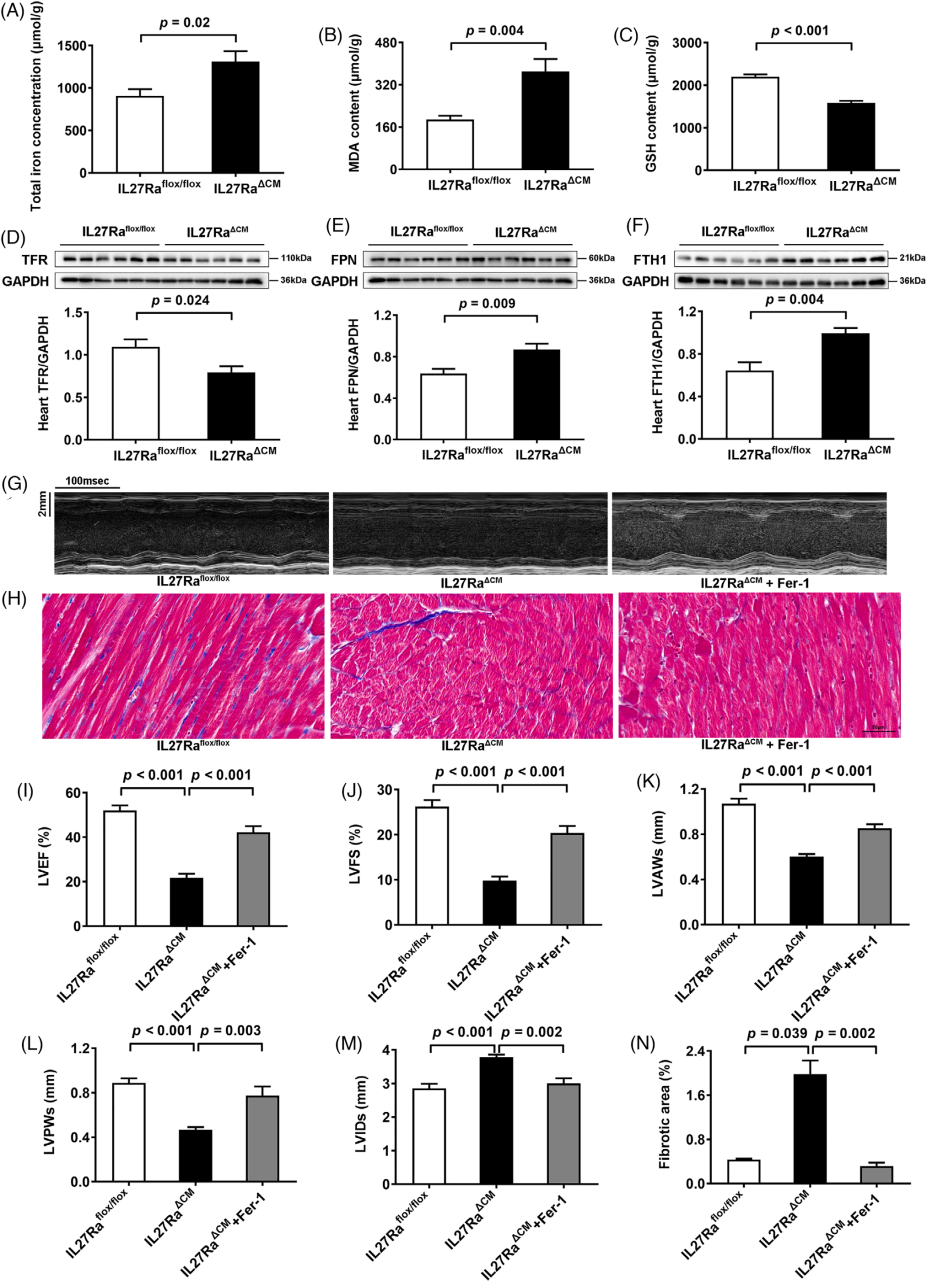
Inhibition of ferroptosis ameliorated DCM in IL27Ra^ΔCM^ mice. (A–C) Total iron, MDA and GSH levels of myocardial tissues in IL27Ra^flox/flox^ and IL27Ra^ΔCM^ groups (*n* = 6). (D–F) Immunoblotting results and quantitative analysis of TFR, FPN and FTH1 protein expression levels in IL27Ra^flox/flox^ and IL27Ra^ΔCM^ groups. (*n* = 6). (G) Representative images of M‐mode echocardiograms. Scale bar = 100 ms/2 mm. (H) Representative images of Masson staining and quantitative analysis of myocardial fibrotic area. Scale bar = 50 µm. (I–M) Quantitative analysis of LVEF, LVFS, LVAWs, LVPWs and LVIDs (*n* = 6). (N) Quantitative analysis of myocardial fibrotic area (*n* = 6). Data are presented as mean ± SEM. MDA: malondialdehyde; GSH: glutathione; TFR: transferrin receptor; FPN: ferroportin; FTH1: ferritin heavy chain 1; GAPDH: glyceraldehyde‐3‐phosphate dehydrogenase; IL27Ra^ΔCM^: cardiomyocyte‐specific IL27 receptor knockout; Fer‐1: ferrostatin‐1; DCM: dilated cardiomyopathy; LVEF: left ventricular ejection fraction; LVFS: left ventricular fractional shortening; LVAWs: left ventricular end‐systolic anterior wall thickness; LVPWs: left ventricular end‐systolic posterior wall thickness; LVIDs: left ventricular end‐systolic internal diameter.

Our study has several limitations. First, our study lacks the validation of in vitro experiments, which could provide additional mechanistic insights. Second, hematopoietic system‐specific IL27Ra conditional knockout mice need to be constructed in future studies to rule out the possibility that the protective effect of IL27 on DCM is independent of immune cells. Third, the therapeutic potential of IL27 supplementation in our mouse model remains to be determined. Fourth, the precise molecular mechanisms by which IL27 regulates ferroptosis need further investigation. Finally, the generalizability of the IL27‐ferroptosis relationship to other DCM models requires additional investigation.

In conclusion, our study demonstrated that IL27 might exert its protective effects in DCM by inhibiting ferroptosis. This finding highlights IL27 as a promising therapeutic target, offering valuable insights for the development of novel treatment strategies aimed at alleviating DCM progression and improving patient prognosis.

## AUTHOR CONTRIBUTIONS

Bing Xiao and Yuming Wu contributed to the conception and design of the study. Yan Zhao and Jing Dai wrote the paper. Yan Zhao, Jing Dai, Angwei Gong, Sheng Jin, Keke Wang, Haijuan Hu, Chengjian Guan and Qianli Ma performed the experiments and analyzed the data. Bing Xiao and Yuming Wu contributed to the critical revision of the manuscript for important intellectual content. All authors reviewed and approved the final manuscript.

## CONFLICT OF INTEREST STATEMENT

The authors declare no conflict of interest.

## FUNDING INFORMATION

This work was supported by the Program for the National Natural Science Foundation of China (32271155 and 91849120), the Project of Hebei Natural Science Foundation (No. H2021206205), the Program for Excellent Talents in Clinical Medicine of Hebei Province (No. ZF2023148) and the S&T Program of Hebei Province (No.22377728D).

## ETHICS STATEMENT

All animal procedures were approved by the Ethics Committee for the Care and Use of Laboratory Animals at the Second Hospital of Hebei Medical University and the protocol for animal experiments followed the Animal Research: Reporting of In Vivo Experiments (ARRIVE) guidelines and performed in compliance with the National Research Council's Guide for the Care and Use of Laboratory Animals.

## Supporting information



Supporting Information

Supporting Information

Supporting Information

Supporting Information

Supporting Information

Supporting Information

## Data Availability

GWAS Data for IL27 is available in the IEU OpenGWAS repository, https://gwas.mrcieu.ac.uk/datasets/prot‐a‐1516/. GWAS Data for DCM is available in the GWAS Catalog repository, https://www.ebi.ac.uk/gwas/publications/32382064. The protein sequence data reported in this paper have been deposited in the Genome Sequence Archive (Genomics, Proteomics & Bioinformatics 2021) in National Genomics Data Center (Nucleic Acids Res 2022), China National Center for Bioinformation / Beijing Institute of Genomics, Chinese Academy of Sciences (GSA: OMIX006288) that are accessible at https://ngdc.cncb.ac.cn/gsa. The data that support the findings of this study during the current study are included within the article (and its supplementary materials files) and available from the corresponding author upon reasonable request.
