# Supplementary material for: Interleukin 27 deficiency drives dilated cardiomyopathy by ferroptosis
Source: Clin Transl Med. 2025 Mar 21;15(4):e70269. doi: 10.1002/ctm2.70269 (PMC11928289; doi:10.1002/ctm2.70269)
Supplement: Supplementary file 1 — Supporting Information [file CTM2-15-e70269-s003.docx]

Supplementary information

**Interleukin 27 deficiency drives dilated cardiomyopathy by ferroptosis**

Yan Zhao ^1,†^, Jing Dai ^2,†^, Angwei Gong ^1^, Sheng Jin ^3^, Chengjian Guan ^1^, Keke Wang ^1^, Qianli Ma ^1^, Haijuan Hu ^1^, Yuming Wu ^3, 4, 5*^, Bing Xiao ^1*^

^1^Department of Cardiology, The Second Hospital of Hebei Medical University, Shijiazhuang 050000, China

^2^Department of Clinical Diagnostics, Hebei Medical University, Shijiazhuang 050017, China

^3^Department of Physiology, Hebei Medical University, Shijiazhuang 050017, China

^4^The Key Laboratory of Neural and Vascular Biology, Ministry of Education, Shijiazhuang 050017, China

^5^Hebei Key Laboratory of Cardiovascular Homeostasis and Aging, Shijiazhuang 050017, China

^†^Yan Zhao and Jing Dai have contributed equally to this work

^*^Correspondence:

Bing Xiao: xiaobing@hebmu.edu.cn

Yuming Wu: wuym@hebmu.edu.cn

1. **Methods**

**1.1 Mendelian randomization analysis**

Mendelian randomization (MR) was an epidemiological approach that employs genetic variants as instrumental variables for a particular exposures to investigate their causal connections with outcomes. In our study, IL27 served as the exposure, and DCM was the outcome. Data for IL27 came from a genome-wide association study (GWAS) comprising of 3301 individuals of European ancestry, while data for DCM came from a GWAS of 35,407 individuals of European ancestry, 95 individuals of African ancestry, 108 individuals of East Asian ancestry, 303 individuals of South Asian ancestry, and 128 individuals of unknown ancestry. SNPs significantly associated with IL27 (*p* < 5 × 10^−8^) were selected and tested for linkage disequilibrium to ensure the independence of SNPs (r^2^ < 0.001; clumping window: 5000kb). Subsequently, we identified these SNPs mentioned above in the outcomes and checked whether these SNPs were associated with the outcomes, and SNPs with a *p* value of less than 5 × 10^−8^ in the outcomes that needed to be eliminated. If no corresponding SNPs could be found in the outcomes, we chose to eliminate those SNPs. The wald ratio method was used for the primary causal estimation, with the significance level set at 0.05, and *p* value less than 0.05 were regarded as statistically significant. The above analysis was performed using the TwoSampleMR (version 0.5.6) software package in R software (version 4.2.1).

- 1. **Animals and treatment**

Male C57BL/6 N mice (10-12 weeks old) were purchased from Vital River Laboratory (Beijing, China); Male IL27 knockout (IL27 KO) mice, cardiomyocyte-specific IL27 receptor knockout (IL27Ra^ΔCM^) mice, genetic wildtype (WT) mice and IL27Ra^flox/flox^ mice (all 10-12 weeks old) were purchased from Cyagen Biosciences Inc (Jiangsu, China). All mice were kept under standard conditions of constant temperature (22°C ± 2°C) and humidity (60%) (12 hours light/dark cycle) with *ad libitum* access to food and water. All animal procedures were approved by the Ethics Committee for the Care and Use of Laboratory Animals at the Second Hospital of Hebei Medical University and the protocol for animal experiments was followed the Animal Research: Reporting of In Vivo Experiments (ARRIVE) guidelines and performed in compliance with the National Research Council's Guide for the Care and Use of Laboratory Animals.

We induced DCM using doxorubicin (Dox), a cardiotoxic agent known to DCM in clinical applications^17^ and widely used in animal experiments.^18,19^ To establish a mouse model of DCM, Dox (HY-15142, MedChemExpress, USA) was administered to mice by a single intraperitoneal injection at a dose of 8 mg/kg on days 0, 7, and 14.^20^ To investigate the role of ferroptosis in DCM, ferrostatin-1 (Fer-1, HY-100579, MedChemExpress, USA), a ferroptosis inhibitor, was administered to animals via daily intraperitoneal injection at a dose of 1 mg/kg for two weeks.^21^ Vehicle controls were treated with an equivalent volume of DMSO diluted in saline to ensure consistency across experimental conditions.

On day 15, following echocardiographic assessment, blood and heart tissues were collected. Blood samples of all mice were centrifuged at 3500 rpm and 4°C for 15 minutes, and plasma was collected and frozen at -80°C. All mouse hearts were fixed with 4% paraformaldehyde, and the left ventricular tissues were rapidly frozen in liquid nitrogen and stored at -80°C for subsequent analyses.

- 1. **Measurement of echocardiography.**

We utilized the Vevo 2100 ultrasound device (Visual Sonics Inc., Toronto, Canada) to obtain transthoracic M-mode echocardiograms. Three consecutive cardiac cycles were captured for each mouse to measure key parameters including left ventricular ejection fraction (LVEF), left ventricular fractional shortening (LVFS), left ventricular end-systolic anterior wall thickness (LVAWs), left ventricular end-systolic posterior wall thickness (LVPWs), and left ventricular end-systolic internal diameter (LVIDs). The average readings for each parameter were recorded.

- 1. **Histological analysis.**

After fixing the left ventricular tissues in 4% paraformaldehyde for 48 hours, they were embedded in paraffin, and consecutive myocardial sections of 4 μm were obtained. Following deparaffinization and hydration, the sections were processed for Masson trichrome staining, and the staining results were observed with a light microscope (Nikon Eclipse CI, Tokyo, Japan). For quantitative analysis of fibrosis, five non-overlapping fields were randomly selected from each section, and the fibrotic area percentage for each field was calculated using ImageJ software (ImageJ v2.0.0, National Institutes of Health, Bethesda, MD, USA). The final result was determined by averaging the percentages from the five analyzed fields.

- 1. **Measurement of IL27 in plasma.**

The enzyme-linked immunosorbent assay (ELISA) kits (KE10016, Proteintech, Illinois, USA) was used to determine the plasma IL27 levels in mice.

**1.6 Measurement of total iron, malondialdehyde (MDA) and glutathione (GSH) levels.**

Heart tissues were harvested and analyzed using kits according to the manufacturer’s protocols to detect the contents of total iron (A039-2-1, Nanjing Jiancheng Bioengineering Institute, Nanjing, China), MDA (S0131S, Beyotime Biotechnology, Shanghai, China) and GSH (A006-2-1, Nanjing Jiancheng Bioengineering Institute, Nanjing, China).

- 1. **Immunoblotting.**

Left ventricular tissues were mechanically homogenized in ice-cold radioimmunoprecipitation (RIPA) buffer, followed by centrifugation at 12,000 rpm for 10 minutes at 4°C. The supernatants were collected for protein extraction, and protein concentrations were quantified using the bicinchoninic acid (BCA) protein assay kit (P0012, Beyotime Biotechnology, Shanghai, China). Subsequently, proteins sample (30 μg) were separated by electrophoresis on 10% sodium dodecyl sulphate polyacrylamide gel electrophoresis and transferred onto PolyVinylideneFluoride membrane (Merck Millipore, Co Cork, Ireland). The membranes were then blocked with 5% non-fat milk at room temperature for 1 hour and then incubated with IL27 antibody (NB100-56389, Novus, Colorado, USA), IL27Ra antibody (ab220359, Abcam, Cambridge, UK), transferrin receptor (TFR) antibody (ab269513, Abcam, Cambridge, UK), ferroportin (FPN) antibody (66606-1-Ig, Proteintech, Illinois, USA), ferritin heavy chain 1 (FTH1) antibody (YT1692, ImmunoWay, Plano, USA) and glyceraldehyde-3-phosphate dehydrogenase (GAPDH) antibody (ET1601-4, Huabio, Hangzhou, China) at 4°C for overnight, followed by incubation with goat anti-mouse secondary antibody (SA00001-1, Proteintech, Illinois, USA) or goat anti-rabbit secondary antibody (5220-0336, Seracare, Massachusetts, USA) at room temperature for 1 hour. The bands were detected using the ChemiDoc XRS+ System (Bio-Rad, California, USA), and grayscale values were quantified with ImageJ software.

- 1. **Agarose gel image of PCR-genotyping of genomic DNA.**

Genomic DNA was extracted from the tissue samples using genomic DNA extraction kit (9765, Takara Bio Inc., Kusatsu, Japan) and amplified by PCR. The PCR products were separated by agarose gel electrophoresis for 30 minutes at constant voltage of 130 V, and finally the gel images were analyzed under ultraviolet light.

- 1. **Quantitative proteomic analysis.**

The heart tissues of 3 samples per group from WT mice, IL27KO mice, IL27Ra^flox/flox^ mice and IL27Ra^ΔCM^ mice were extracted and their protein concentrations were measured. Subsequently, the proteins were subjected to tandem mass tagging (TMT) labeling, followed by reverse-phase chromatography separation. The samples were then analyzed against a database, and finally underwent bioinformatics analysis. Detailed methods can be found in the supplementary materials.

- 1. **Statistical analysis.**

Statistical analysis was conducted with SPSS 21.0 software, presenting numerical data as mean ± SEM. The differences between two groups were assessed using a two-tailed t-test, while comparisons among three groups were conducted using one-way ANOVA, with a significance level set at *p* < 0.05.

1. **Detailed methods for Quantitative Proteomic Analysis**

**2.1 Method for Quantitative Proteomic Analysis**

**2.1.1 Protein extraction**

Take out the frozen sample, add liquid nitrogen, grind fully, and take out the appropriate amount into 1.5mL centrifuge tube/2mL high-speed centrifuge tube. Add the sample lysate, add phosphatase inhibitor, protease inhibitor PMSF to make the final concentration of 1mM. The cold lapping machine grinds at -35°C, 60Hz, 120s, once again. The solution was centrifuged at 12000rpm at 4℃ for 10min to obtain the supernatant, and then centrifuged again to obtain the supernatant. The supernatant is the total protein solution of the sample, and the protein concentration is determined and stored at -80°C.

**2.1.2 Protein digestion**

According to the measured protein concentration, take the same quantity protein from each sample, and dilute different groups of samples to the same concentration and volume. Add 25mm DTT of the corresponding volume into the above protein solution to make the DTT final concentration about 5mM, and incubate at 55°C for 30-60min. Then add the corresponding volume of iodoacetamide so that the final concentration was about 10mm, and place it in the dark for 15-30min at room temperature. Then 6 times of the volume of precooled acetone in the above system to precipitate the protein, and place it at -20°C for more than four hours or overnight. After precipitation, take out the sample and centrifuge at 8000g for 10min at 4°C for collecting the precipitate. According to the amount of protein, add the corresponding volume of enzymolysis diluent (protein: enzyme = 50:1 (m/m), 100μg of protein add 2μg of enzyme) to redissolve the protein precipitate, then the solutions were incubated for digestion at 37°C for 12h. Finally, samples were lyophilized or evaporated after enzymolysis.

**2.1.3 Label**

For TMT labelling, the lyophilized samples were resuspended in 30μL 100mM TEAB and Labeling reaction in a 1.5mL Ep tube. 20μL acetonitrile were added to TMT reagent vial at room temperature. The centrifuged reagents were dissolved for 5min and mixed for centrifugation and repeat this step once. Then 10μL of the TMT label reagent was added to each sample for mixing. The tubes were incubated at room temperature for 1h. Finally, 5µL of 5% hydroxylamine were added to each sample and incubated for 15min to terminate reaction. The labeling peptides solutions were lyophilized and stored at -80°C.

**2.1.4 Liquid chromatography-mass spectrometry**

RP separation was performed on an 1100 HPLC System (Agilent) using an Agilent Zorbax Extend-C18 column (2. 1×150 mm, 5 μm). Mobile phases A (ACN-H2O (2:98, v/v), adjust pH to 10 with ammonia) and B (ACN-H2O (90:10, v/v), adjust pH to 10 with ammonia) were used for RP gradient. The solvent gradient was set as follows: 0~8 min, 98% A; 8~8.01 min, 98%~95% A; 8.01~48 min, 95%~75% A; 48~60 min, 75~60% A; 60~60.01 min, 60~10% A; 60.01~70 min, 10% A; 70~70.01 min, 10~98% A; 70.01~75 min, 98% A. Tryptic peptides were separated at an fluent flow rate of 300 μL/min and monitored at 210nm. Samples were collected for 8-60 minutes, and eluent was collected in centrifugal tube 1-15 every minute in turn. Samples were recycled in this order until the end of gradient. The separated peptides were lyophilized for mass spectrometry. All analyses were performed by a Q Exactive HF mass spectrometer (Thermo, USA) equipped with a Nanospray Flex source (Thermo, USA). Samples were loaded and separated by a C18 column (15cm × 75µm) on an EASY-nLCTM 1200 system (Thermo, USA). The flow rate was 300nL/min and linear gradient was 75min (0~4 min, 8-11% B; 4~36 min, 11-45% B; 36~39 min, 45-100% B; 39~45 min, 100% B. mobile phase A = ACN-H2O-FA (99.9:0.1, v/v) and B = ACN-H2O-FA (99.9:0.1, v/v)). The first stage MS quality resolution is set to 45,000, the automatic gain control value is set to 3e6, and the maximum injection time is 30ms. MS scanning was set to the full scanning charge mass ratio m/z range of 350-1500, and MS/MS scanning was performed on 25 of the highest peaks. All MS/MS spectra were collected using high-energy collision cracking in data-dependent positive ion mode, and the collision energy was set to 32. The resolution of MS/MS is set to 15000, the automatic gain control is set to 2e5, and the maximum ion injection time is 30ms. The dynamic exclusion time is set to 30 seconds.

**2.1.5 Database search**

ProteomeDiscoverer (v.2.4) was used to search all of the raw data thoroughly against the Uniprot Mus Musculus database. Database search was performed with Trypsin digestion specificity. Alkylation on cysteine was considered as fixed modifications in the database searching. For protein quantification method, TMT was selected. A global false discovery rate (FDR) was set to 0.01 and protein groups considered for quantification required at least 1 peptide.

**2.1.6 Statistical analyses**

The thresholds of fold change (>1.2 or <0.83) and P-value <0.05 were used to identify differentially expressed proteins (DEPs).

**2.2 Primer details**

**2.2.1 Primers sequence for IL27 PCR-genotype identification**

| Primers1 forward primer (F1) | 5’-GTCCCTAGAATAGAACCACTGGC-3 ’ |
| --- | --- |
| Primers1 reverse primer (R1) | 5’-GTGCTGTAAACGACGGGACTTTCTC-3 ’ |
| Primers2 forward primer (F2) | 5’-CAGAATCAGGTTGAAGAAGCCTAG-3’ |
| Primers2 reverse primer (R1) | 5’-GTGCTGTAAACGACGGGACTTTCTC-3 ’ |

Primers1 product size: 534 bp

Primers2 product size: 472 bp

**2.2.2 Primers sequence for IL27Ra PCR-genotype identification**

| locus of X-over P1 (loxP) forward primer | 5’-GTCCCTAGAATAGAACCACTGGC-3 ’ |
| --- | --- |
| loxP reverse primer | 5’-GTGCTGTAAACGACGGGACTTTCTC-3 ’ |
| Cre recombinase forward primer | 5’-CAGAATCAGGTTGAAGAAGCCTAG-3’ |
| Cre recombinase reverse primer | 5’-GTGCTGTAAACGACGGGACTTTCTC-3 ’ |

loxP primers product size:

Homozygotes: one band with 217 bp

Heterozygotes: two bands with 217 bp and 148 bp

Wildtype: one band with 148 bp

Cre recombinase primers product size: 530 bp

1. **Information for supplementary figure**

**
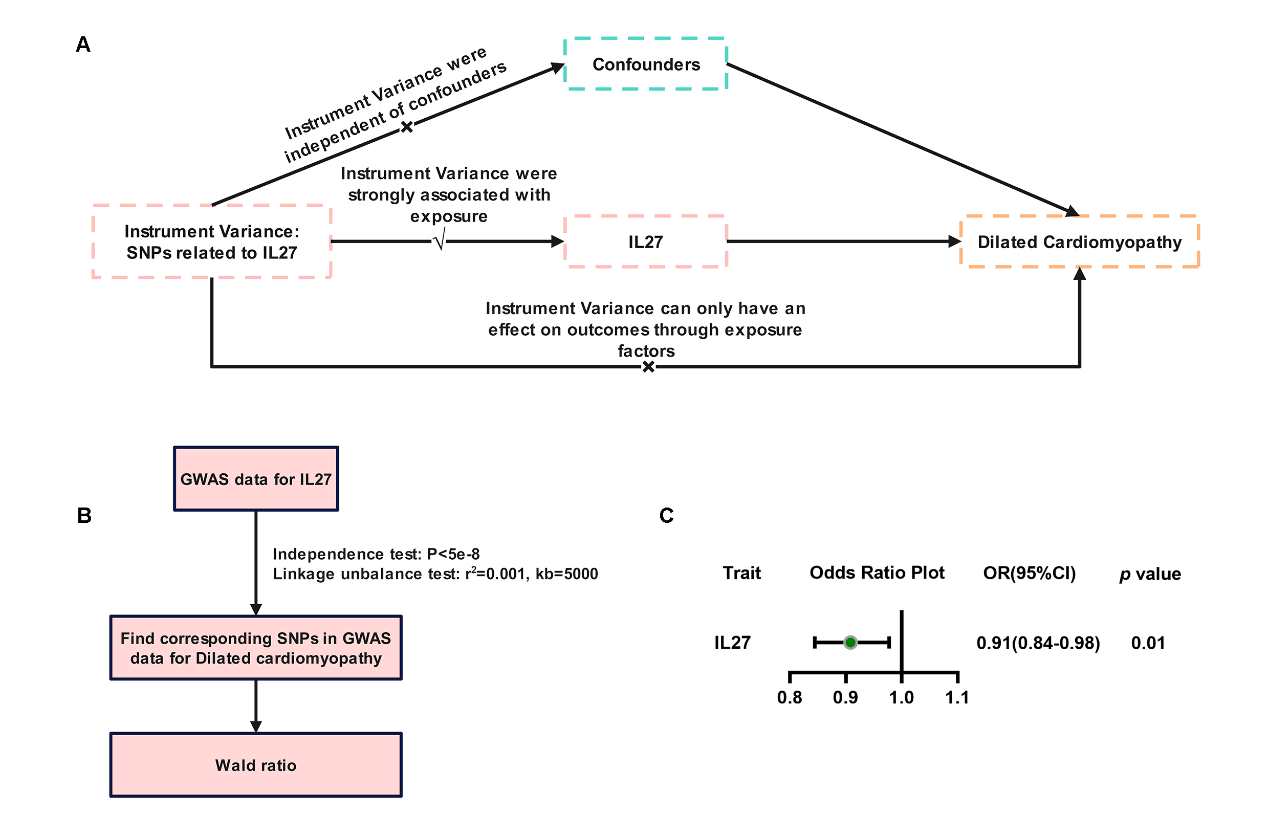
**

**Supplementary** **Fig. 1 MR analysis suggested that IL27 was associated with DCM.** (A) The three fundamental principles of the MR method. (B)The general process of MR analysis in this study. (C) Causal effect value between IL27 and DCM as shown by the wald ratio method. MR: mendelian randomization; IL27: interleukin 27; DCM: dilated cardiomyopathy.


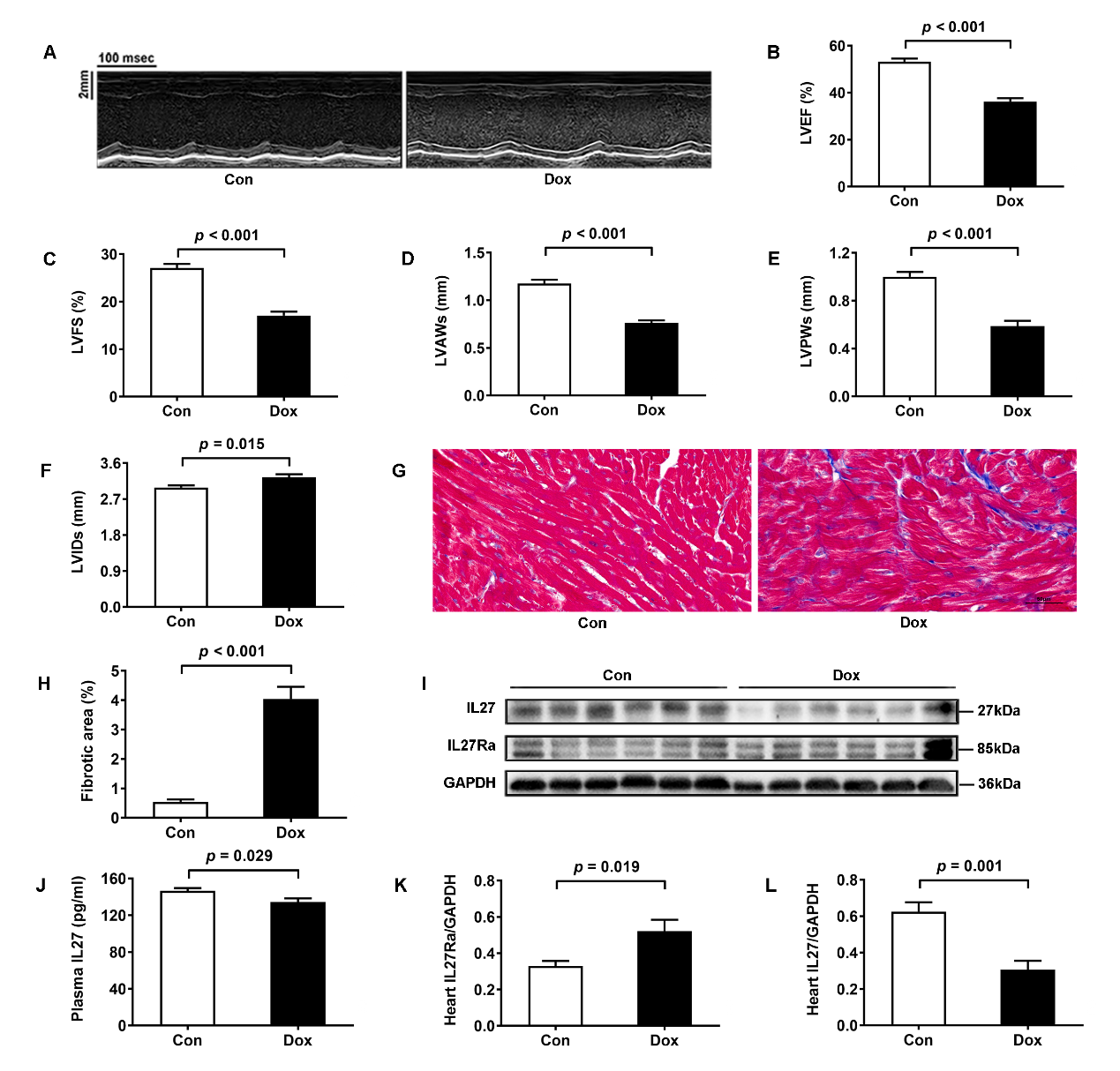


**Supplementary** **Fig. 2 IL27 was down-regulated in Dox-treated mice.** Mice were treated with 8 mg/kg Dox on days 0, 7 and 14 to establish a DCM model. (A) Representative images of M-mode echocardiograms on day 15. Scale bar = 100 ms/2 mm. (B-F) Quantitative analysis of LVEF, LVFS, LVAWs, LVPWs and LVIDs (n=8). (G-H) Representative images of Masson staining and quantitative analysis of myocardial fibrotic area (n=8). Scale bar = 50 μm. (I) Immunoblotting results of IL27 and IL27Ra in mice heart tissues. GAPDH was used as normalization control. (J) Quantitative analysis of plasma IL27 levels determined by ELISA (n=8). (K-L) Quantitative analysis of protein expression levels of IL27Ra and IL27 in heart tissues (n=6). Data are presented as mean ± SEM. Con: control; IL27: interleukin 27; IL27Ra: interleukin 27 receptor; Dox: doxorubicin; DCM: dilated cardiomyopathy; LVEF: left ventricular ejection fraction; LVFS: left ventricular fractional shortening; LVAWs: left ventricular end-systolic anterior wall thickness; LVPWs: left ventricular end-systolic posterior wall thickness; LVIDs: left ventricular end-systolic internal diameter. GAPDH: glyceraldehyde-3-phosphate dehydrogenase; ELISA: enzyme-linked immunosorbent assay.


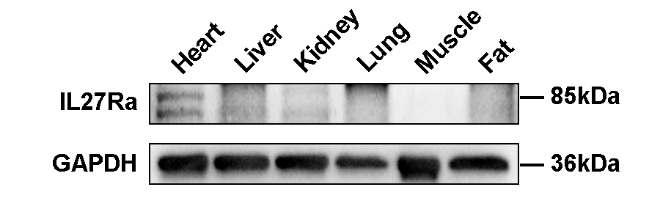


**Supplementary Fig. 3 Expression of IL27Ra in various tissues of mice.** IL27Ra: interleukin 27 receptor; GAPDH: glyceraldehyde-3-phosphate dehydrogenase.


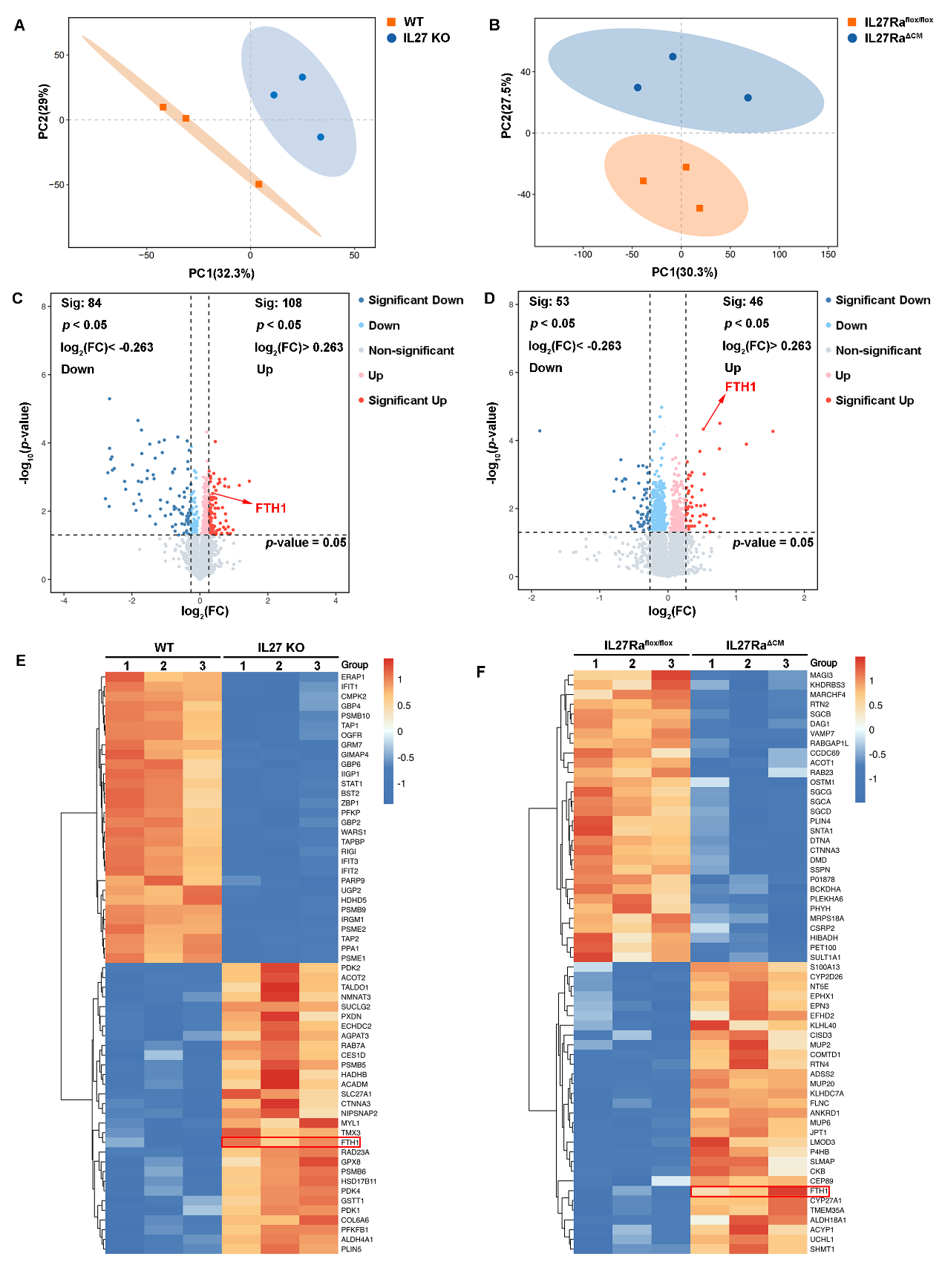


**Supplementary Fig. 4 Proteomic evidence revealed ferroptosis as a potential mechanism by which IL27 regulated DCM.** (A) PCA in WT and IL27 KO groups (n=3). (B) PCA in IL27Ra^flox/flox^ and IL27Ra^ΔCM^ groups (n=3). (C) Volcano plot of differentially expressed proteins between WT and IL27 KO groups. *p* < 0.05, fold change < 0.73 or fold change > 1.2 were significantly different proteins. (D) Volcano plot of differentially expressed proteins between IL27Ra^flox/flox^ and IL27Ra^ΔCM^ groups. *p* < 0.05, fold change < 0.73 or fold change > 1.2 were significantly different proteins. (E) Heatmap showing differential proteome expression between WT and IL27 KO groups. (F) Heatmap showing differential proteome expression between IL27Ra^flox/flox^ and IL27Ra^ΔCM^ groups. Data are presented as mean ± SEM. WT: wildtype; IL27 KO: interleukin 27 knockout; IL27Ra^ΔCM^: cardiomyocyte-specific IL27 receptor knockout; DCM: dilated cardiomyopathy; FTH1: ferritin heavy chain 1.


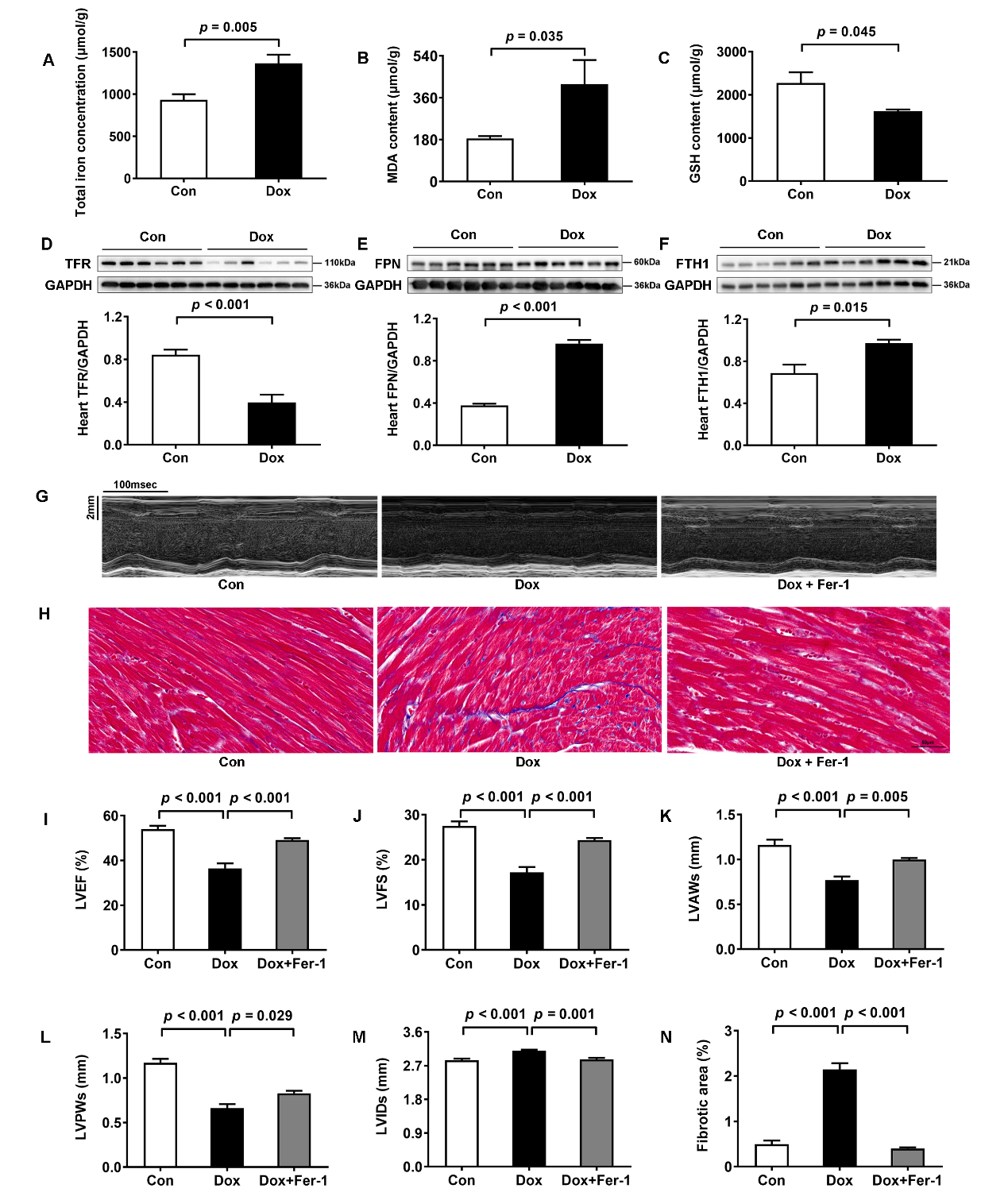


**Supplementary Fig.5 Inhibition of ferroptosis ameliorated DCM in DOX-treated mice.** (A-C) Total iron, MDA and GSH levels of myocardial tissues in Con and Dox groups (n=6). (D-F) Immunoblotting results and quantitative analysis of TFR, FPN, FTH1 protein expression levels in Con and Dox groups. (n=6). (G) Representative images of M-mode echocardiograms. Scale bar = 100 ms/2 mm. (H) Representative images of Masson staining and quantitative analysis of myocardial fibrotic area. Scale bar = 50 μm. (I-M) Quantitative analysis of LVEF, LVFS, LVAWs, LVPWs and LVIDs (n=6). (N) Quantitative analysis of myocardial fibrotic area (n=6). Data are presented as mean ± SEM. Con: control; Dox: doxorubicin; MDA: malondialdehyde; GSH: glutathione; TFR: transferrin receptor; FPN: ferroportin; FTH1: ferritin heavy chain 1; GAPDH: glyceraldehyde-3-phosphate dehydrogenase; Fer-1: ferrostatin-1; DCM: dilated cardiomyopathy; LVEF: left ventricular ejection fraction; LVFS: left ventricular fractional shortening; LVAWs: left ventricular end-systolic anterior wall thickness; LVPWs: left ventricular end-systolic posterior wall thickness; LVIDs: left ventricular end-systolic internal diameter.

1. **Declation**

**4.1 Ethics approval and consent to participate**

All animal procedures were approved by the Ethics Committee for the Care and Use of Laboratory Animals at the Second Hospital of Hebei Medical University and the protocol for animal experiments was followed the Animal Research: Reporting of In Vivo Experiments (ARRIVE) guidelines and performed in compliance with the National Research Council's Guide for the Care and Use of Laboratory Animals.

**4.2 Data availability statement**

GWAS Data for IL27 is available in IEU OpenGWAS repository, https://gwas.mrcieu.ac.uk/datasets/prot-a-1516/. GWAS Data for DCM is available in GWAS Catalog repository, https://www.ebi.ac.uk/gwas/publications/32382064. The protein sequence data reported in this paper have been deposited in the Genome Sequence Archive (Genomics, Proteomics & Bioinformatics 2021) in National Genomics Data Center (Nucleic Acids Res 2022), China National Center for Bioinformation / Beijing Institute of Genomics, Chinese Academy of Sciences (GSA: OMIX006288) that are accessible at https://ngdc.cncb.ac.cn/gsa. The data that support the findings of this study during the current study are included within the article (and its supplementary information files) and available from the corresponding author on reasonable request.

**4.3 Funding**

This work was supported by the Program for the National Natural Science Foundation of China (32271155 and 91849120), the Project of Hebei Natural Science Foundation (No. H2021206205), the Program for Excellent Talents in Clinical Medicine of Hebei Province (No. ZF2023148) and the S&T Program of Hebei Province (No. 22377728D).

**4.4 Author's contributions**

Bing Xiao and Yuming Wu contributed to the conception and design of the study. Yan Zhao and Jing Dai wrote the paper. Yan Zhao, Jing Dai, Angwei Gong, Sheng Jin, Keke Wang, Haijuan Hu, Chengjian Guan, Qianli Ma performed the experiments and analyzed the data. Bing Xiao and Yuming Wu contributed to the critical revision of the manuscript for important intellectual content. All authors reviewed and approved the final manuscript.
