# Supplementary figures and images for: Interleukin 27 deficiency drives dilated cardiomyopathy by ferroptosis
Source: Clin Transl Med. 2025 Mar 21;15(4):e70269. doi: 10.1002/ctm2.70269 (PMC11928289; doi:10.1002/ctm2.70269)

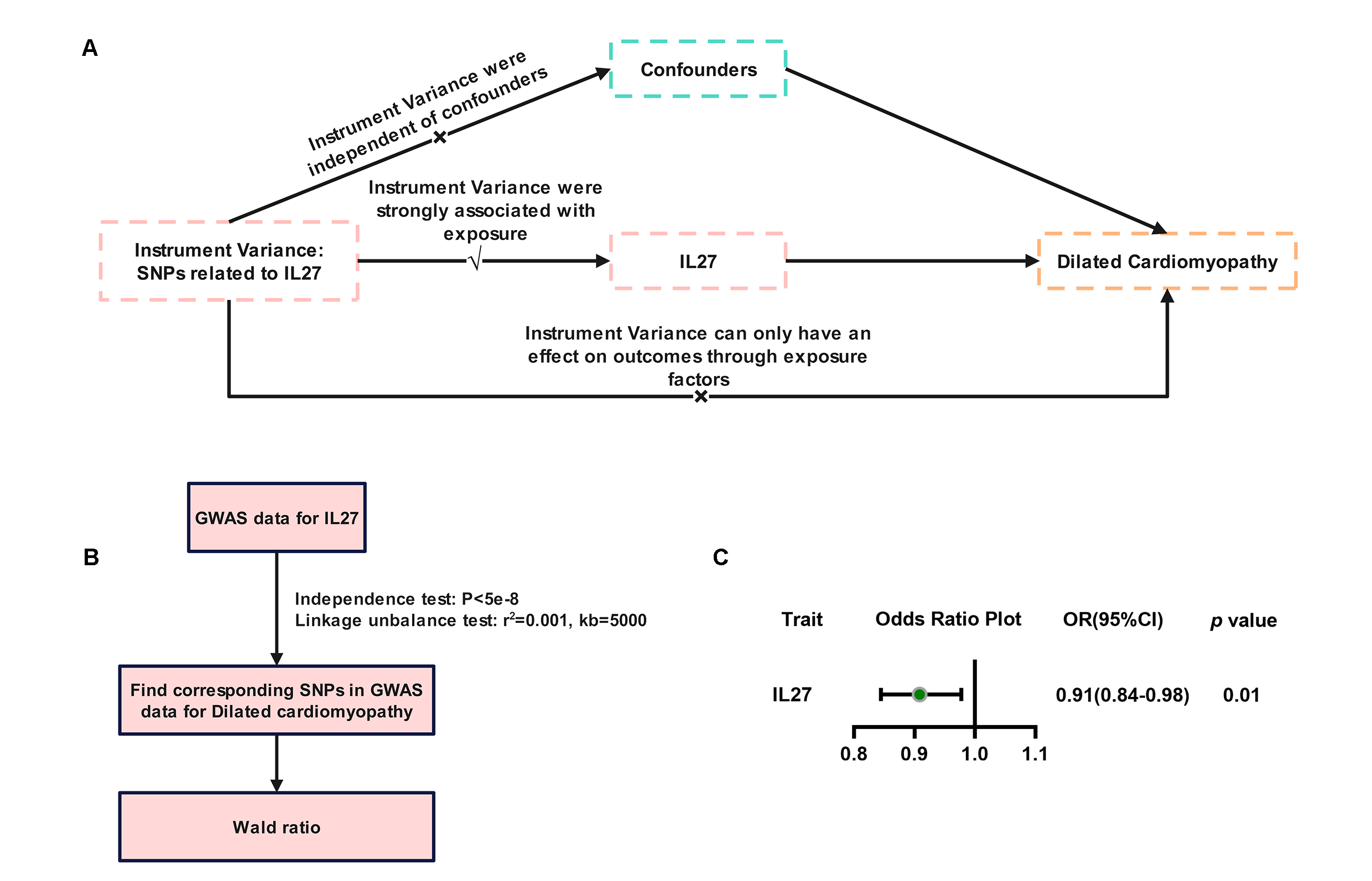

Supplement: Supplementary file 2 — Supporting Information [file CTM2-15-e70269-s002.tif]

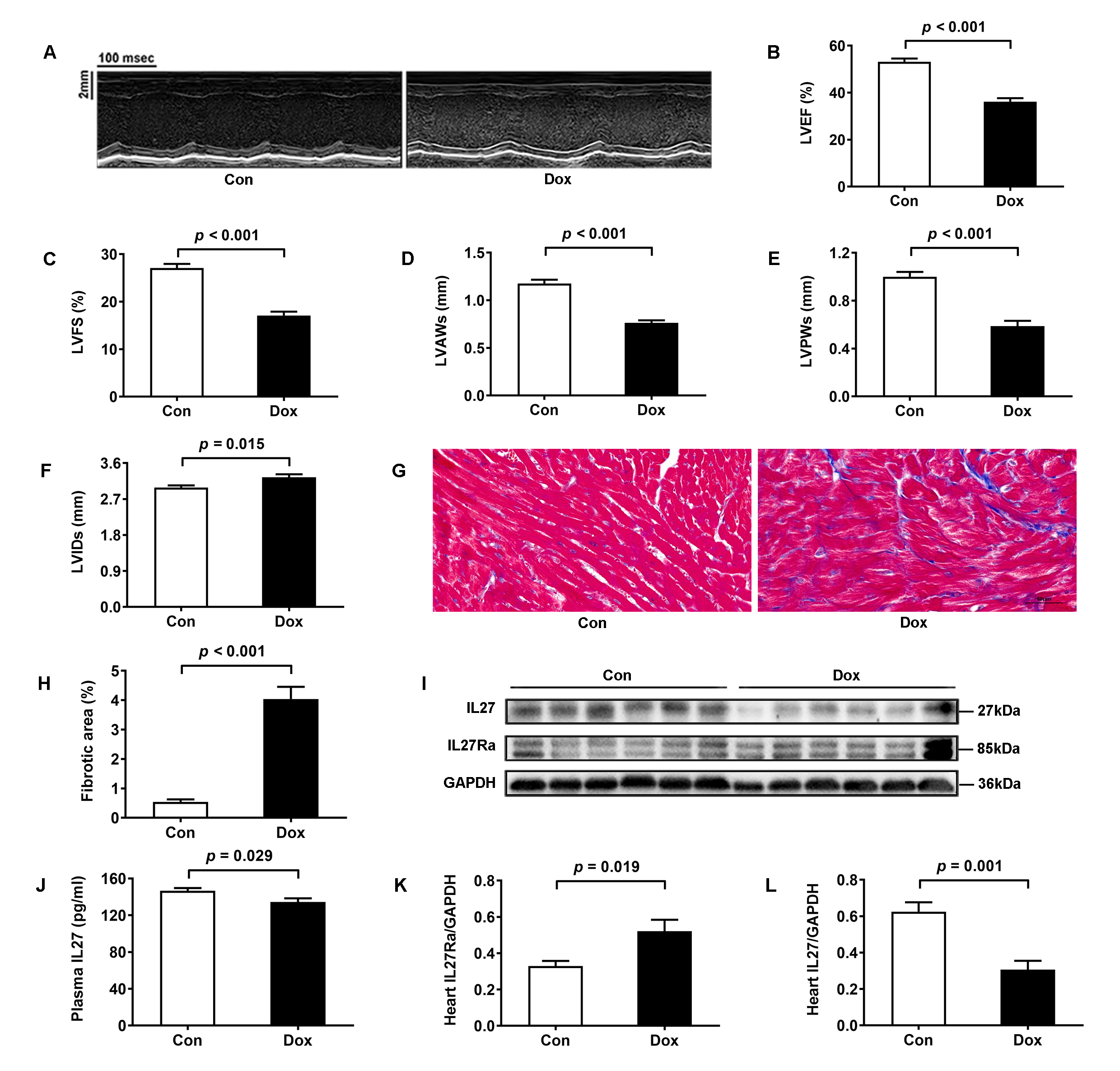

Supplement: Supplementary file 3 — Supporting Information [file CTM2-15-e70269-s001.tif]

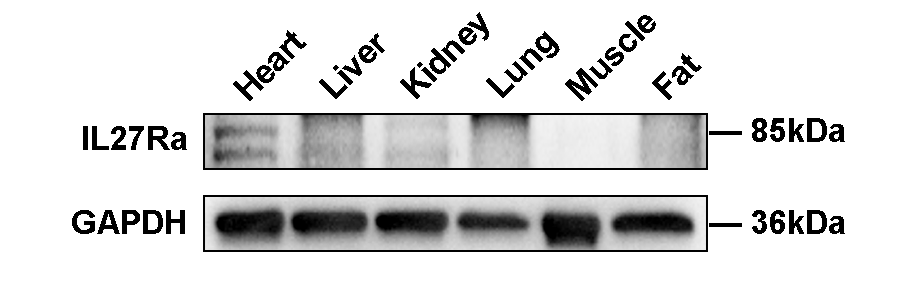

Supplement: Supplementary file 4 — Supporting Information [file CTM2-15-e70269-s005.tif]

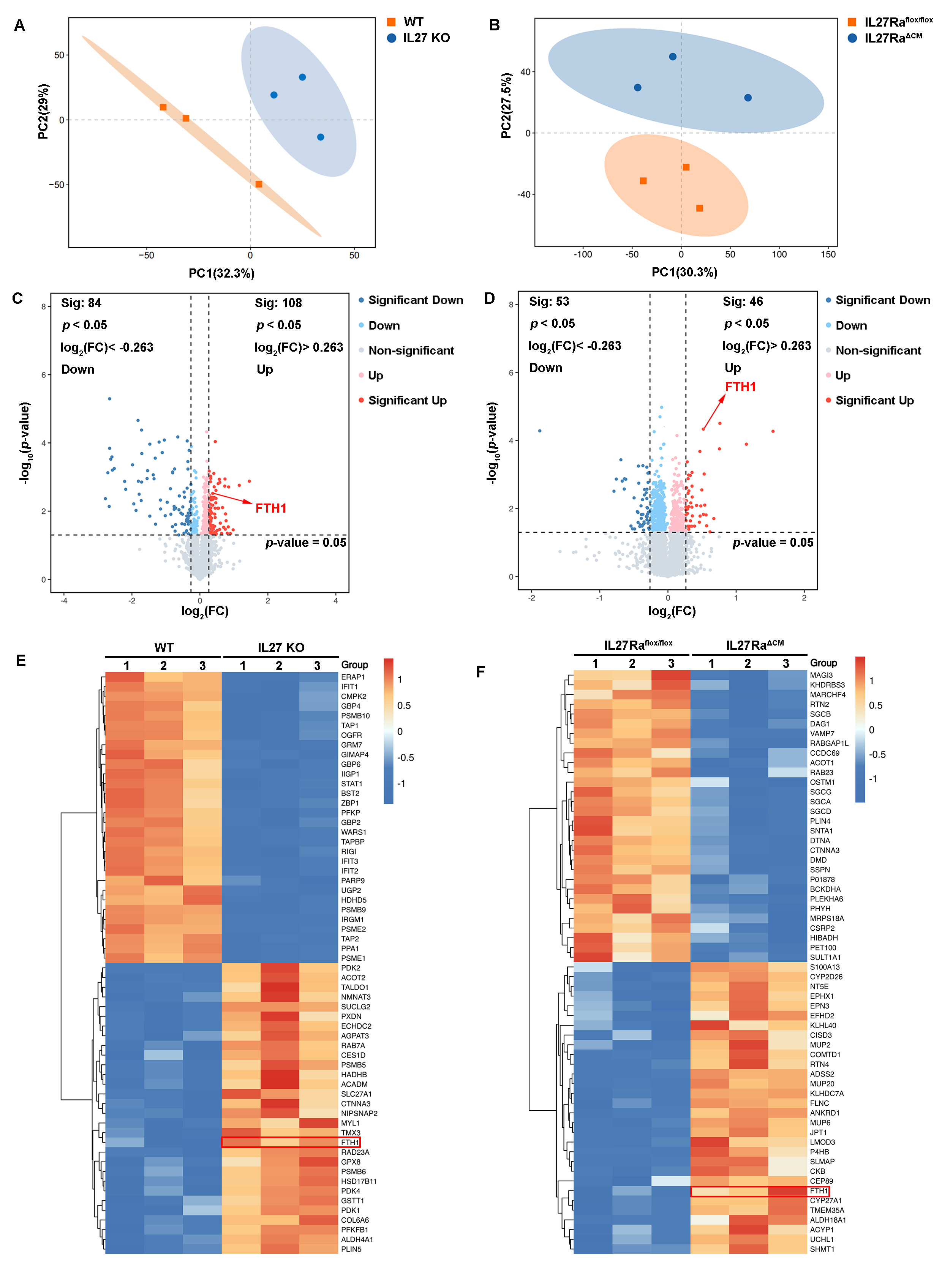

Supplement: Supplementary file 5 — Supporting Information [file CTM2-15-e70269-s004.tif]

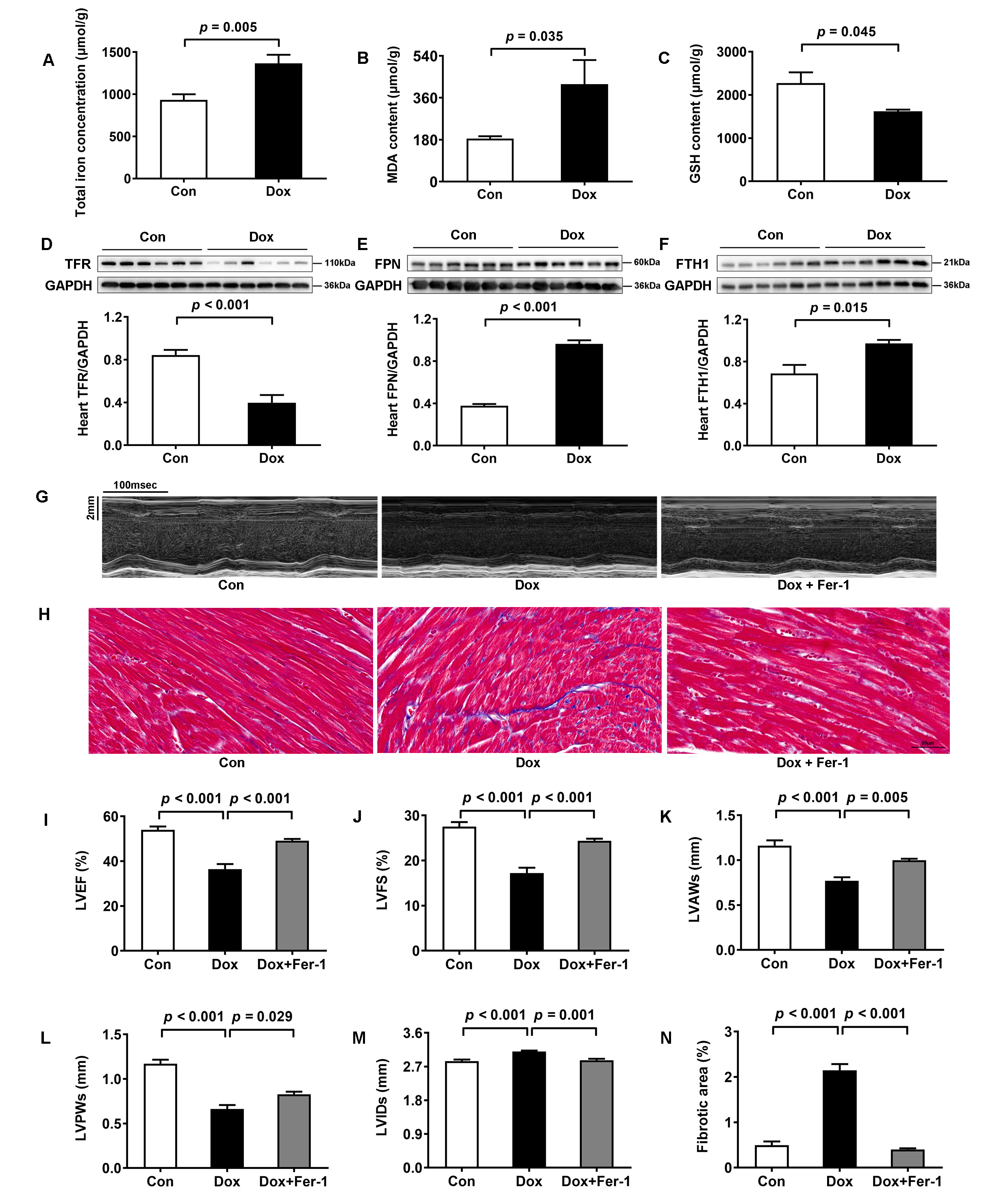

Supplement: Supplementary file 6 — Supporting Information [file CTM2-15-e70269-s006.tif]
